# Why not just Google it? An assessment of information literacy skills in a biomedical science curriculum

**DOI:** 10.1186/1472-6920-11-17

**Published:** 2011-04-25

**Authors:** Karl Kingsley, Gillian M Galbraith, Matthew Herring, Eva Stowers, Tanis Stewart, Karla V Kingsley

**Affiliations:** 1 Department of Biomedical Sciences, School of Dental Medicine, University of Nevada, Las Vegas, Las Vegas, Nevada, USA; 2 University Libraries, University of Nevada, Las Vegas, Las Vegas, Nevada, USA; 3 Office of Academic Assessment, School of Dental Medicine, University of Nevada, Las Vegas, Las Vegas, Nevada, USA; 4 Department of Information Technology, School of Dental Medicine, University of Nevada, Las Vegas, Las Vegas, Nevada, USA; 5 Instructional Systems Design, Federal Deposit Insurance Corporation, Corporate University, Arlington, Virginia, USA; 6 College of Education, Teacher Education Department, University of New Mexico, Albuquerque, New Mexico, USA

## Abstract

**Background:**

Few issues in higher education are as fundamental as the ability to search for, evaluate, and synthesize information. The need to develop information literacy, the process of finding, retrieving, organizing, and evaluating the ever-expanding collection of online information, has precipitated the need for training in skill-based competencies in higher education, as well as medical and dental education.

**Methods:**

The current study evaluated the information literacy skills of first-year dental students, consisting of two, consecutive dental student cohorts (n = 160). An assignment designed to evaluate information literacy skills was conducted. In addition, a survey of student online search engine or database preferences was conducted to identify any significant associations. Subsequently, an intervention was developed, based upon the results of the assessment and survey, to address any deficiencies in information literacy.

**Results:**

Nearly half of students (n = 70/160 or 43%) missed one or more question components that required finding an evidence-based citation. Analysis of the survey revealed a significantly higher percentage of students who provided incorrect responses (n = 53/70 or 75.7%) reported using Google as their preferred online search method (p < 0.01). In contrast, a significantly higher percentage of students who reported using PubMed (n = 39/45 or 86.7%) were able to provide correct responses (p < 0.01). Following a one-hour intervention by a health science librarian, virtually all students were able to find and retrieve evidence-based materials for subsequent coursework.

**Conclusions:**

This study confirmed that information literacy among this student population was lacking and that integration of modules within the curriculum can help students to filter and establish the quality of online information, a critical component in the training of new health care professionals. Furthermore, incorporation of these modules early in the curriculum may be of significant value to other dental, medical, health care, and professional schools with similar goals of incorporating the evidence base into teaching and learning activities.

## Background

Although many critical issues face both faculty and students in higher education, few of these issues are as critical to academic success as the interconnected skills and capabilities required to search for and retrieve, as well as evaluate and synthesize various types of information, a process more commonly known as information literacy [1,2]. Educated consumers of information need the technological skills to navigate library resources, online search engines, and metasearch tools. The spectrum of information literacy, however, extends beyond the paradigm of skills-based computer literacy [3] and simple find-and-report interpretations of online searches. Whether it is freely available on the Web or accessible through restricted databases, online information from a vast array of sources must be scrutinized for credibility, reliability, timeliness, and applicability for performing a specific task or solving a problem. Additionally, evaluative skills are needed to interpret data from multiple sources, including printed text, statistics, symbolic representations, maps, charts, tables, and still and moving images [4].

The emerging networked technologies comprising the participatory Web, also known as Web 2.0, have profoundly changed the way information is produced, distributed, and consumed [5-8]. Wikis, blogs, pod casts, video sharing, social networking sites, and other online applications offer innumerable opportunities for user generated content (UGC) and information sharing through what has been called an "architecture of participation" [9]. Although these new participatory technologies provide rich opportunities for information sharing, they also pose new challenges for information seekers. Torrents of unfiltered information are uploaded to, and downloaded from, the Internet every day. In addition, users generate, remix, repurpose, store, and then share this digital information. As a result, Web users must continually balance the need for easy to find, readily available, reliable information and to avoid questionable, inaccurate, incomplete or deceptive online information.

The proliferation of online data has precipitated the need for more curricular activities that teach and require students to effectively search for and evaluate information in higher education and health care settings. Few studies have examined how effectively university-level students evaluate and use online information. A recent university-led study found the majority of participants began their searches with, and preferred using, Google as compared to the instructor-recommended databases for information problem solving [10]. A study by this group found a majority (54%) of dental students (n = 78) in a biomedical science course were unable to locate primary research and evidence-based references, despite specific and detailed instructor directions to use PubMed [1]. These studies demonstrate that online searches of university-level students are primarily facilitated through the use of Google and not through other evidence-based research tools [11-17]. The overwhelming popularity of Google as the first and primary search tool preferred by university students inevitably raises the question: "Why not just Google it?" To date, researchers have not provided an adequate response to this question.

Based upon this information, the current study evaluated the information literacy skills of first-year dental students. This study also surveyed the preferred methods of online information searches and database usage of these students. Finally, demographic characteristics were examined, including age, sex, or race which might correlate with the use of particular search engines or the need for improved information literacy competencies. These findings would then be incorporated into the curriculum by means of an intervention designed by the University Health Sciences Librarian. The goal of this intervention would be to improve student performance, address any questions regarding the quality and reliability of online information sources, and to provide specific instructions for finding evidence-based materials for subsequent use.

## Methods

### Participants

The study design consisted of a clustered convenience sample within a dental school setting, which took place over two consecutive years. More specifically, dental students from two consecutive cohorts (C1, n = 84, C2, n = 76) enrolled in a first-year dental course at the University of Nevada, Las Vegas - School of Dental Medicine were given an assignment designed to evaluate information literacy skills (ILS). In brief, students were given a review article of vaccines against dental caries (tooth cavity formation) from 2001 [18] and were then asked to provide answers related to content (technology-independent). In addition, students were also asked to use a specific web-based, online technology (PubMed) to find more recently published, peer-reviewed citations (technology-dependent) (Appendix 1). No students were excluded from participation. All students completed the assignment for a response rate of 100% (n = 160).

### Human Subjects Exemption

All students from both dental student cohorts (n = 160) completed both the assignment and survey, and were included in this study. Student assessment data for this assignment were retrieved and each record was assigned a numerical, non-duplicated identifier to prevent disclosure and ensure confidentiality of personally identifiable private information. Gender, age, and race were noted separately for each student record, in separate tables, prior to assignment to provide demographic information.

This protocol was reviewed by the UNLV Biomedical Institutional Research Board (IRB) and was deemed excluded from IRB review (OPRS#0811-2911). Informed Consent was waived pursuant to the exemption to human subjects research under the Basic HHS Policy for Protection of Human Research Subjects, (46.101) Subpart A (b) regarding IRB Exemption for (2) research involving the use of education tests (cognitive, diagnostic, aptitude, achievement) where the subjects cannot be identified or linked, directly or through identifiers, to the individual subjects.

### Assessment and Evaluation

Three questions, which addressed separate aspects of fundamental knowledge, were divided into two parts, A and B. Part A of each question was content specific. Obtaining full credit was based solely upon the students' ability to list or define the correct response(s). Part B of each question involved using a web-based technology to search for citations to support their answers to Part A. Obtaining full credit was based upon the students' ability to comprehend the overall task, translate and apply this knowledge, evidenced by the citation. Parts A and B were scored separately, as correct or incorrect, and responses tallied.

### Survey - Needs Assessment

All students were given a Needs Assessment, designed by the Office of Academic Assessment and the Director of Information Technology, which was administered in conjunction with the assignment described above.

Five questions asked students to: 1. Identify where they were likely to seek evidence-based information first, 2. Rank various databases and search engines in order of preference and usage, 3. Identify any databases or search engines used that were not previously listed, 4. Identify the most helpful or useful databases and search engines, and 5. Identify the least helpful or most difficult databases and search engines (Appendix 2).

### Statistical evaluation

Cronbach's alpha was used to determine the internal consistency of the Needs Assessment survey. At least one-fifth of students from each cohort (C1, n = 19; C2, n = 15) were re-tested one week after the initial survey in order to gauge reliability. Virtually all responses were unchanged (C1 = 94.7%, n = 18/19; C2 = 100%, n = 15/15). The reliability coefficient was calculated to be 0.92, which indicated a high internal consistency for this instrument.

Following the assessment of information literacy skills, hypothesis testing could be performed using a chi-square (χ^2^) test to determine if any characteristic (demographic or search preference) was different than expected among any specific group of students. Students who missed one or more sections of the technology-dependent portion of the assignment could be tested to determine if the proportion of those with any particular characteristic (gender, age, race, search preference) falls outside of the range that could reasonably be expected. A probability level of alpha (α) = 0.05 was used to determine significance.

### Intervention

Based upon the results of the survey (Needs Assessment) and the student assignment scores, an intervention was designed and implemented in collaboration with the Health Sciences Librarian. The one-hour intervention provided specific training to establish what defines the evidence base. Other topics included how to ascertain if information has been peer-reviewed. Hands-on training to utilize the library website and database search engines was given, as well as demonstrations of simple and advanced searching methods using PubMed and MESH.

## Results

### Assessment and Evaluation

**Figure 1:**
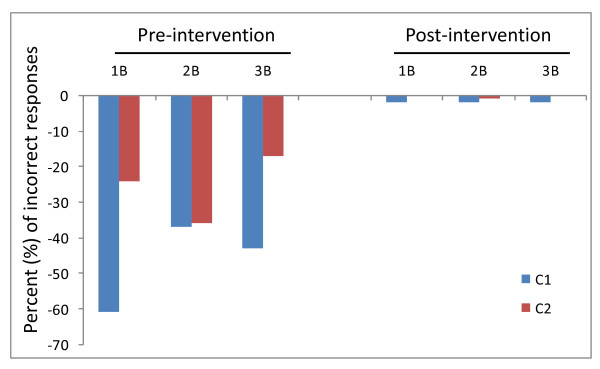
**Results of an information literacy skills (ILS) assessment from two consecutive dental school cohorts (C1, C2)**. A majority of students from the first cohort (C1) missed at least one part of the technology-dependent ILS component of the assignment questions (1B = 61% incorrect, n = 51/84; 2B = 37% incorrect, n = 31/84; 3B = 43% incorrect, n = 36/84). Similarly, more than one-third of students from the subsequent cohort (C2) also missed one or more sections of ILS-related assignment questions (1B = 24% incorrect, n = 18/76; 2B = 36% incorrect, n = 27/76; 3B = 17% incorrect, n = 13/76).

Evaluation of the student assignments revealed that the vast majority (>90%) of students were able to demonstrate their knowledge of the content-specific, technology-independent portion of each question (1A, 2A, 3A; data not shown). However, more than half of students from the first cohort (C1) were unable to provide an evidence-based citation to demonstrate their proficiency with at least one of the three online, technology-dependent ILS portions (1B, 2B, 3B) of the assignment (Figure 1). Similarly, between one-third and one-half of students from the second cohort (C2) in the subsequent year were also unable to locate and provide an evidence-based citation to at least one of the three ILS components of this assignment.

### Demographics

To assess whether any characteristics were associated with an incorrect response, demographic information for each cohort was collected and evaluated (Table 1). In general, students from both cohorts were in their mid- to late-twenties (25.4 years old, on average), were mostly male (63%) and white (66%). The average age of students who scored one or more incorrect responses (25.8 years old) was not significantly different than expected (χ^2 ^= 0.132, p > 0.10). In addition, the proportion of incorrect respondents who were males (68.5%) was not significantly different (χ^2 ^= 0.645, p > 0.50). However, the percentage of respondents with incorrect responses who were white was significantly higher (80%, n = 56/70) than expected (χ^2 ^= 3.869, p < 0.05).

**Table 1:** Demographic characteristics of respondents

	Cohort (C1) n = 84	Cohort (C2) n = 76	Total (C1+C2) n = 160
Age	25.1 y, STD = 2.2	26.2 y, STD = 3.9	25.4 y, STD = 2.4
Sex	Male, n = 47 (56%)	Male, n = 54 (71%)	Male, n = 101 (63%)
	Female, n = 37 (44%)	Female, n = 22 (29%)	Female, n = 59 (37%)
Race	White, n = 55 (65%)	White, n = 50 (66%)	White, n = 105 (66%)
	Other, n = 29 (35%)	Other, n = 26 (34%)	Other, n = 55 (34%)
	Asian, n = 23 (27%)	Asian, n = 18 (24%)	Asian, n = 41 (26%)
	Hispanic, n = 4 (5%)	Hispanic, n = 6 (8%)	Hispanic, n = 10 (6%)
	Black, n = 1 (1%)	Black, n = 2 (3%)	Black, n = 3 (2%)
	Am. Ind., n = 1 (1%)	Am. Ind., n = 0 (0%)	Am. Ind., n = 1 (1%)
			

Incorrect respondents	Cohort (C1)	Cohort (C2)	Total (C1+C2)

Age	25.5 y, STD = 2.6	26.0 y, STD = 3.1	25.8 y, STD = 2.8
Sex	n = 36/51 M (70.6%)	n = 12/19 M (63.2%)	n = 48/70 M (68.5%)
	n = 15/51 F (29.4%)	n = 7/19 F (36.8%)	n = 22/70 F (31.4%)
Race	n = 46/51 W (90.2%)	n = 10/19 W (52.6%)	n = 56/70 W (80%)*
	n = 5/51 O (9.8%)	n = 9/19 O (47.4%)	n = 14/70 O (20%)*
	Asian, n = 3/51 (6%)	Asian, n = 8/19 (42%)	Asian, n = 11/70 (16%)
	Hisp., n = 1/51 (2%)	Hisp., n = 0 (0%)	Hisp., n = 1/70 (1.4%)
	Black, n = 1/51 (2%)	Black, n = 1/19 (5%)	Black, n = 2/70 (3%)
	Am. Ind., n = 0 (0%)	Am. Ind., n = 0 (0%)	Am. Ind., n = 0 (0%)

* denotes p < 0.05; ** denotes p < 0.01

M = Male, F = Female, W = White, O = Other, Hisp. = Hispanic, Am. Ind. = American Indian or Native American, y = years, STD = standard deviation

### Needs Assessment Survey

To determine the preferred methods of web-based searches and database utilization among these students, a Needs Assessment survey was also administered, in conjunction with the assignment (Table 2). Google was cited most frequently in both cohorts (C1 = 47.6%; C2 = 36.8%) as the site they were most likely to seek evidence-based information from first. Google was also ranked the highest choice among the databases and search engines listed, in order of preference and usage within both cohorts. Although PubMed was the second most commonly cited search engine (C1 = 29.7%; C2 = 26.3%), this represented only slightly more than one quarter of all respondents (28.1%, n = 45/160). Wikipedia was ranked third, with other choices (Google Scholar, Medline, Medscape, CINAHL) less frequently listed.

**Table 2:** Results of Needs Assessment

Preferred Database or Search Engine	Cohort (C1) n = 84	Cohort (C2) n = 76	Total (C1+C2) n = 160
Google	n = 40 (47.6%)	n = 28 (36.8%)	n = 68 (42.5%)
PubMed	n = 25 (29.7%)	n = 20 (26.3%)	n = 45 (28.1%)
Wikipedia	n = 11 (13.1%)	n = 16 (21.1%)	n = 27 (16.9%)
Google Scholar	n = 4 (4.8%)	n = 9 (11.8%)	n = 13 (8.1%)
Medline	n = 2 (2.4%)	n = 1 (1.3%)	n = 3 (1.9%)
Medscape	n = 0 (0.0%)	n = 2 (2.6%)	n = 2 (1.3%)
CINAHL	n = 2 (2.4%)	n = 0 (0.0%)	n = 2 (1.3%)
			

Additional or Not Listed Databases/Search Engines	Frequency of responses:		
	C1 n = 84	C2 n = 76	Total n = 160

Yahoo	15	4	19 (11.9%)
SciVerse Scopus	9	5	14 (8.8%)
Library website	8	2	10 (6.3%)
Facebook, YouTube, etc.	8	2	10 (6.3%)
EBSCO	0	0	0 (0%)
Academic Search Premier	0	5	5 (3.1%)
ScienceDirect, WebMD	0	3	3 (1.9%)
None/Not Listed	44	56	100 (62.5%)
			

Most Helpful or Useful	C1	C2	Total

Google	44	22	66 (41.3%)
Wikipedia	17	21	38 (23.8%)
PubMed	11	15	26 (16.3%)
Other (Medline, Scopus)	10	14	24 (15.0%)
Not Listed	2	4	6 (3.8%)
			

Most Difficult or Least Helpful	C1	C2	Total

PubMed	43	26	69 (43.1%)
None/Not Listed	21	24	45 (28.1%)
Other (MSN, Yahoo)	12	8	20 (12.5%)
Other (Medline, Medscape)	7	9	16 (10.0%)
Library website	1	9	10 (6.3%)

Among the databases and search engines that students found most helpful or useful, Google was listed by more than 40% of all respondents as their top choice (42.5%, n = 68/160) (Table 2). Wikipedia was the next most commonly cited by students as most helpful, by nearly one-fourth of respondents (23.8%, n = 38/160). When asked which databases and search engines students found most difficult and least helpful, PubMed was the only selection that more than one fifth of students listed (43.1%, n = 69/160).

To assess whether student preferences of online search engines were associated with an incorrect response, results of the Needs Assessment were also compiled and evaluated (Table 3). Of those students with one or more incorrect responses, more than three-quarters (75.7%) listed Google as their preferred search engine. This was significantly greater than would be expected (χ^2 ^= 31.586, p < 0.01). Although the proportion of students with incorrect responses listing Wikipedia as their preference (14.3%) was not different (χ^2 ^= 0.345, p > 0.10), significantly fewer students with incorrect answers listed PubMed (10%) than would be expected (χ^2 ^= 11.350, p < 0.01).

**Table 3:** Analysis of survey responses and assessment scores.

Incorrect respondents	Cohort (C1)	Cohort (C2)	Total (C1+C2)
Google	n = 40/51 (92.2%)	n = 13/19 (68.4%)	n = 53/70 (75.7%)**
Wikipedia	n = 8/51 (15.7%)	n = 2/19 (10.5%)	n = 10/70 (14.3%)
PubMed	n = 3/51 (5.9%)	n = 4/19 (21.1%)	n = 7/70 (10.0%)**
			

Correct respondents	Cohort (C1)	Cohort (C2)	Total (C1+C2)

Google	n = 0/40 (0.0%)	n = 15/28 (53.6%)	n = 15/68 (22.1%)**
Wikipedia	n = 3/11 (27.3%)	n = 8/16 (50.0%)	n = 11/27 (40.7%)
PubMed	n = 22/25 (88.0%)	n = 17/20 (85.0%)	n = 39/45 (86.7%)**

* denotes p < 0.05; ** denotes p < 0.01

To provide further analysis, results of the Needs Assessment were examined for any associations among students providing correct responses (Table 3). Of those students with correct responses, significantly fewer (22.1%) cited Google as their preferred search engine (or listed Google as most helpful) than expected (χ^2 ^= 29.130, p < 0.01). Conversely, the proportion of students with correct responses who listed PubMed as their first search preference (86.7%) was significantly higher than expected (χ^2 ^= 10.336, p < 0.01).

Based upon the results of the survey (Needs Assessment), an intervention was designed and implemented in collaboration with the Health Sciences Librarian. Following this one-hour intervention, a similar assignment was administered and virtually all students were able to provide evidence-based references and citations to the technology-dependent portions of each question (C1: 1B, 2B, 3B = 98% correct; C2: 1B, 3B = 100% correct; 2B = 99% correct) (Figure 1).

## Discussion

This study sought to evaluate information literacy among two cohorts of first-year, dental students, as well as to assess their preferred methods of conducting online searches. This information was then evaluated with respect to student performance. These results strongly suggested that a large percentage of entering, first-year dental students have neither the skills nor the training to locate, evaluate, and retrieve evidence-based information, supporting previous observations from this group [1]. More importantly, this study is among the first to evaluate what search engines and databases these students preferred and how helpful, useful, or difficult they ranked each of these technologies. Finally, this study provided evidence that suggested student preference for the use of Google to search for information was more closely associated with an incorrect response than any other search engine or database evaluated.

These findings suggest that many of the links, references, and data returned by Google searches are not appropriate for use in an evidence-based curriculum. Although the survey found that students clearly prefer to utilize Google, either based upon familiarity or ease of use [10], the references and citations many of the students reported were, in fact, advertisements, promotional materials, foreign media sources, and even personal websites (including one Facebook page reference). These findings provide an impetus for faculty and administrators to recognize that training and skills-based experience with online resources may be lacking, even among university-level students. These data now suggest some initial answers to the question "Why not just Google it?"

Interestingly, student preference for PubMed was strongly associated with the ability to demonstrate ILS. Although some variability was evident between the two cohorts examined, student preference for PubMed was less likely to be associated with an incorrect response in both cohorts. Moreover, preference for PubMed was clearly evident among students with correct responses in both cohorts (>85%).

One promising result from this study was that a simple, well-constructed, one-hour intervention led by an ILS library specialist, may be sufficient to effectively help students. This included information on how to perform online information searches, how to assess the quality and reliability of databases, and detailed instruction to find information that is evidence-based and academically appropriate. These findings also suggest the need for placing this type of curricular instruction very early in a medical- or graduate-level health sciences curriculum, to ensure that all students, regardless of their background and academic training, have equal opportunities for success in their educational programs.

Previous studies of information literacy found some associations between race and gender. These data may suggest these factors might limit access to computers, internet access, and research-based coursework for female and minority students [19]. However, the current study found that although minorities comprised a relatively large percentage of the overall student cohort population (47.4%), they represented a disproportionately small percentage of students who were unable to complete the assignment (20%). Although it is possible that recent University-wide initiatives to address disparities and access among college-level females and minorities may be responsible for these results, other limitations of the study design must also be considered relevant. For example, although previous studies have found that age was often a significant factor [20], this study used a convenience sample of dental students that was biased toward relative youth (average age = 25.4 years old) and found no such association.

### Study limitations

Due to the study design of consecutive dental student cohorts, the sample size and diversity of the student populations evaluated was somewhat limited. In addition, this study was conducted at a public institution, which may have also had multiple interconnected effects on the types of students who applied, and were admitted, to this particular institution.

No data regarding income and socioeconomic status were available, which limited some of the potential analysis and conclusions that could be inferred from this study. Low socioeconomic status may have the potential to limit student access to online tools and technologies. Based upon this information, future study designs might include these variables to more accurately evaluate the potential influence on student performance. Additionally, future studies might also be of longer duration to help reduce the effects of any year-to-year variability.

## Conclusions

The goals of this study were to evaluate information literacy of first year dental students, assess their preferred methods of conducting online searches, and to find any associations between these findings. This study confirmed that information literacy was lacking and, importantly, that many students prefer to use Google - despite specific and detailed instructions otherwise. Preference for the use of Google was significantly associated with students who were unable to find evidence-based citations. This deficit was remedied with a targeted one-hour intervention facilitated an ILS librarian. These results suggest that integration of the evidence-base into teaching and learning modules within a health sciences curriculum can help students to filter and establish the quality of online information [21]. Furthermore, incorporation of these modules early in the curriculum may be of significant value to other dental, medical, health care, and professional schools with similar goals of incorporating and integrating the evidence base into teaching and learning activities.

## Competing interests

The authors declare that they have no competing interests.

## Authors' contributions

KK, GMG, and KVK conceived and coordinated this project. GMG was course director for the courses used in this study. KK was responsible for administering the student assignments. TS and KK were responsible for developing and administering the needs assessment. ES was responsible for administering the intervention. KK and KVK were responsible for the analysis and interpretation of the data. MH and GMG made significant contributions to the editing of this manuscript. All authors have read and approved the final version of this manuscript.

## Appendix 1

Caries Immunology and Vaccine Research Assignment

Course: Oral Pathogens and Oral Immunology

Summary:

Dental caries is among the most prevalent diseases affecting human populations. Homeostatic changes of the normal oral bacterial ecology, and an overgrowth of specific bacteria, such as *Streptococcus mutans*, are the primary causal factors associated with the formation of caries lesions. Most dental treatments target the elimination of this caries-causing bacterium - although more recent strategies have been designed to prevent the colonization of *S. mutans *and other bacterium through vaccination.

Assignment:

Given the following review article, Michalek, Katz and Childers A Vaccine against Dental Caries. *BioDrugs *(2001) 15 (8): 501-508, summarize briefly your answer to the following questions (1-3):

1. A. Is dental caries infectious? B. Provide at least one citation or reference from the evidence base that supports your answer to part A of this question.

2. A. Which oral microorganism(s) is/are associated with caries? B. Provide at least one citation or reference that supports your answer to part A of this question.

3. A. What are the virulence factors of this/these organism(s)? B. Provide at least one citation or reference that supports your answer to part A of this question.

4. Given that this article is almost nine years old, provide an updated bibliography

Instructions:

Go to PubMed at the following URL: http://www.ncbi.nlm.nih.gov/sites/entrez

Use appropriate search terms (i.e. caries, vaccines, immunology)

a. **Find and list at least five (5) articles **specific to caries vaccine design or development that were published more recently than Michalek, Katz and Childers (2001).

b. For one (1) of these articles, briefly summarize the vaccination strategy (active, passive), the immunogen (adhesin, etc.) and the results (did it work?).

Objectives and Outcomes:

1. Upon completion of this exercise, the student will be able to discuss biomedical science concepts of caries immunology and caries vaccines in the context of oral health and disease;

2. The student will be able to critically evaluate relevant primary scientific literature regarding caries immunology and caries vaccines;

3. The student will be able to review and build an updated bibliography of current literature regarding caries vaccines.

## Appendix 2

DS1 Student Needs Assessment

1. You need to find information for an assignment that includes citations, references and the evidence base. How would you be ***most***likely to search for that information first

A. CINAHL

B. Google

C. Google scholar

D. Medline

E. Medscape

F. PubMed

G. Wikipedia

2. Please rank these database/search engines in order of how often you access each for clinical, pre-clinical, biomedical or professional studies assignments and information.

(1 = most often, 2 = often ...7 = least often/rarely/never)

__________CINAHL

__________Google

__________Google scholar

__________Medline

__________Medscape

__________PubMed

__________Wikipedia

3. Please list which databases or search engines that you use which were not listed above.

4. A. Please list which databases or search engines that you find most helpful or useful.

B. Please describe the features that make this database/search engine useful/helpful to you.

5. A. Please list any databases or search engines that you find difficult to use or access.

B. Please describe any features that make this database/search engine difficult to use/access.

## Pre-publication history

The pre-publication history for this paper can be accessed here:

http://www.biomedcentral.com/1472-6920/11/17/prepub
